# Role of Adult Asthma Education in Improving Asthma Control and Reducing Emergency Room Utilization and Hospital Admissions in an Inner City Hospital

**DOI:** 10.1155/2017/5681962

**Published:** 2017-05-04

**Authors:** Rashmi Mishra, Muhammad Kashif, Sindhaghatta Venkatram, Teresa George, Kristina Luo, Gilda Diaz-Fuentes

**Affiliations:** ^1^Division of Pulmonary and Critical Care, Bronx-Lebanon Hospital Center, Bronx, NY, USA; ^2^Department of Pharmacy, Bronx-Lebanon Hospital Center, Bronx, NY, USA

## Abstract

*Objective*. Asthma education programs have been shown to decrease healthcare utilization and improve disease control and management. The purpose of our study was to evaluate the impact of an outpatient adult asthma education program in an inner city hospital caring for patients with low socioeconomic and educational status.* Methods*. An asthma education program was implemented in September 2014. Patients who received education from September 2014 to July 2015 were evaluated. Outcomes were compared for the same group of patients before and after education. Primary outcomes were emergency room (ER) visits and hospital admissions. Secondary outcomes were change in Asthma Control Test (ACT) score and number of pulmonary clinic visits.* Results*. Asthma education significantly decreased number of patients requiring ER visits and hospital admissions (*p* = 0.0005 and *p* = 0.0015, resp.). Asthma control as per ACT score ≥ 20 improved with education (*p* = 0.0001) with an increase in clinic visits (*p* = 0.0185).* Conclusions*. Our study suggests that implementation of a structured asthma education program in an inner city community hospital has a positive impact on reduction of ER visits and hospital admissions with improvement in asthma control. Institutional Review Board Clinical Study registration number is 01081507.

## 1. Introduction

According to the National Health Interview Survey of 2014, approximately 30 million people had been diagnosed with asthma in their lifetime. 17.7 million (7.4%) adults still have asthma; out of these, around 7 million (43.6%) suffered from asthma attacks in 2014 [1]. The number of patients with asthma increased by 28% from 2001 to 2011 [2]. The estimated total cost of asthma to society was $56 billion (2009 dollars) in 2007. This included medical expenses, loss of productivity resulting from missed school or work days, and premature death [2].

The Global Initiative for Management and Prevention of Asthma (GINA) guidelines were developed to improve the care and control of the disease [3]. Asthma education is recommended for all children and adults as part of management [3]. Multiple studies have shown that education improves outcomes like asthma-related emergency room (ER) utilization and hospitalization, unscheduled physician visits, days off work, and quality of life (QoL) [4]. Studies showing improvement in QoL and decreased ER utilization and hospitalization utilized multiple education sessions over few months [4–7].

Low socioeconomic status is associated with asthma and respiratory symptoms [8]. Inadequate home characteristics, exposure to environmental tobacco smoke, air pollution, and sustained exposure to indoor and outdoor pathogens are some factors that have been associated with development of respiratory diseases in this population [9–14]. Lack of asthma education as a contributor to the poor control of asthma in an inner city population has not been clearly described. Bronx county in NYC ranks lowest among all 62 counties in New York state in all of the following domains: health outcomes, 62/62, health factors, 62/62, clinical care, 62/62, social and economic factors, 62/62 (high school, 60%; some college, 50%), and physical environment, 62/62. [15]. This county has large minority and immigrant populations. Demographics are distinct with large minority and immigrant populations with low socioeconomic status, as indicated by education, employment, and homelessness rates. The prevalence of asthma in this group is one of the highest in the country. Bronx-Lebanon Hospital Center (BLHC) is in South Bronx and serves a large population with asthma. We aimed to evaluate the role of an asthma education program in the control of asthma in our inner city patient population.

## 2. Materials and Methods

This was a pre-and-post study of all asthmatic patients managed in the pulmonary clinic. The study period was from September 2013 to July 2016. An asthma education program was implemented in September 2014 and includes on-site certified asthma educators.

We included all asthmatic patients that participated in structured asthma education from September 2014 to July 2015 and had clinic follow-up until July 2016.

**Figure 1:**
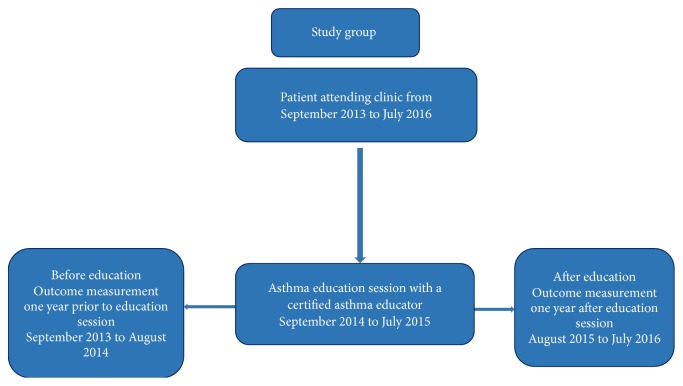
Study design.

Outcomes were compared for the same group of patients during the preeducation period (September 2013 to August 2014) and the posteducation period (August 2015 to July 2016) (Figure 1).

Approval for this study was obtained from the Institutional Review Board (IRB) of Bronx Lebanon Hospital (IRB # 01081507).

### 2.1. Primary Outcomes Included

Primary outcomes included the following:

1. Number of patients requiring ER visits during the preeducation and posteducation periods
2. Number of patients requiring hospital admission during preeducation and posteducation periods. In addition to ER or hospitalization at our institution, we included patients' reports of other ER visits or hospitalization due to asthma.

### 2.2. Secondary Outcomes Included

Secondary outcomes included the following:

1. Change in asthma control assessed by the Asthma Control Test (ACT) score at visit following the asthma education
2. Number of pulmonary clinic visits during preeducation and posteducation periods

Multiple ER visits or admissions were defined as more than one ER visit or hospitalization for asthma during the study period.

### 2.3. Structure of the Asthma Education Program

The asthma educators included two on-site certified asthma educators, a bilingual (English and Spanish) respiratory therapist and a clinical pharmacist. The education was done in the patient's preferred language with help of interpreter services, if required. Translator phone services are available.

The education sessions consisted of a personalized (one-to-one) 30-minute sessions with an asthma educator.

Documentation in the electronic medical record (EMR) followed a structured asthma education note. This form captures data including demographics, ACT score, medications, adherence with medications, and asthma action plan.

The education sessions usually followed the visit to the pulmonologist.

On-site spirometry was performed as per the discretion of the physician.

All patients received written education material available in English and Spanish. Those materials were created following national guidelines and included basic definition of asthma, disease management and control, medications pictures and descriptions, role of peak flow, and environmental control and trigger.

The goals of the education session consisted of having the patient understand the following:

1. What is asthma?
2. Disease management and control
3. Adequate and appropriate use of inhalers, spacers, and peak flow meter
4. Review of written educational materials
5. Asthma action plan

At the end of the session, patients were required to be able to demonstrate appropriate use of inhalers and peak flow meter.

### 2.4. Statistical Analysis

The analysis utilizes preeducation and posteducation data. The sample population of this study consisted of a total of 231 patients. Only those patients who were followed up at clinic one year prior to and one year after education were included. Institutional medical records were reviewed to assess number of hospitalization incidences, ER visits, asthma control score, and asthma severity in the preintervention and postintervention periods. We evaluated documentation including patient reporting ER or hospital admissions to other institutions. Patients who were lost to follow-up after intervention were excluded.

IBM SPSS v19.0 was used for statistical analysis.

The number of patients requiring ER visits and hospitalization were calculated using unpaired *t*-tests. The average numbers of ER visits and hospitalization incidences were calculated using “Compare means” analysis of IBM SPSS. The effect of asthma education on asthma control evaluated using ACT score was compared with the help of unpaired *t*-test. The number of clinic visits was compared using unpaired *t*-tests.

## 3. Results

### 3.1. Demographic Data

250 patients participated in initial education; 19 patients were excluded as they were lost in follow-up. 231 patients were analyzed.  Table 1 shows the baseline characteristics of the patients.

Distribution of the severity of asthma was as follows: 2.6% intermittent, 23.8% mild, 53.7% moderate, and 19.9% severe persistent. At least a third of our patients had comorbid conditions: psychiatric illness followed by gastroesophageal reflux and obesity were the most common (Table 1). Anxiety and depression accounted for majority of psychiatric diseases.

132 (56.7%) of the 231 patients were given an asthma action plan during education. 50 patients (22%) received more than one education session, 42 (18%) received two sessions, 9 (4%) received three sessions, and one (0.4%) received four education sessions.

### 3.2. Primary Outcomes

During the post-asthma-education period, there was a significant decrease in the number of patients requiring ER visits as compared to preeducation period [preeducation (*n* = 94) and posteducation (*n* = 60); *p* = 0.0005, 95% CI]. Asthma education also reduced the number of patients requiring hospitalization [preeducation (*n* = 49) and posteducation (*n* = 26); *p* = 0.0015, 95% CI].

We also found a significant decrease in the average number of ER visits per patient/year [0.91 (preeducation) to 0.61 (posteducation) (*p* = 0.04, 95% CI)] and in the average number of hospital admissions per patient/year [0.34 (preeducation) to 0.19 (posteducation) (*p* = 0.0015, 95% CI)].

A subset evaluation of patients with more than one ED visit or hospital admission showed no impact of asthma education (Table 2).

### 3.3. Secondary Outcomes

The effect of education on asthma control was evaluated using an ACT score ≥ 20 as per GINA guidelines. Patients without preeducation and posteducation ACT score were excluded from analysis. 126 (55%) patients had ACT score available. Asthma education had a statistically significant (*p* = 0.0001, 95% CI) effect on the number of asthmatics with ACT score ≥ 20 (*n* = 20, 15.8% before education, versus *n* = 44, 34.9% after education) (Table 2).

There was a change in number of clinic visits during the preeducation and posteducation periods [1.76 (preeducation) versus 2.18 (posteducation) (*p* = 0.01, 95% CI)].

## 4. Discussion

In this study, we found that a comprehensive asthma education program in an inner city asthmatic group decreased hospital and ER utilization and improved asthma control as assessed by the ACT score. To our knowledge, this is one of the first studies using a short education session incorporated to the same day clinic visit.

Improved asthma outcomes with education have been reported in other socially deprived populations like ours. De Oliveira et al. conducted a study in Brazil to evaluate the effectiveness of asthma education. 53 asthmatic patients were studied: 26 in the education and 27 in the control group. Monthly education provided for six months resulted in decreased ER visits and better symptoms control and scores on QoL questionnaire; however, there was no reduction in hospital admissions [7]. We attempted to eliminate potential environmental confounders and triggers that arise when comparing different patients; so we compared same patients, assuming that triggers and leaving conditions remained stable.

One of the purposes of personalized and structured education is to highlight the importance of each patient's ethnic and socioeconomic background. The presence of inadequate health literacy has not been associated with difficulty in learning or retaining inhaler technique [16]. Asthma education should be tailored to the specific ethnic groups considering cultural differences. In a study done in UK among different ethnic groups of white Europeans and Indians, a significant decrease in physician visit, hospitalization, and use of corticosteroids and antibiotics occurred in only the white European population but not in Indians [17]. In another study among Chinese and Punjabis, an improvement in knowledge of asthma symptoms, inhaler use, and understanding of physician's instructions over 3 months after asthma education is reported. The program included watching education videos or reading educational pamphlets [18]. In our program, interpreter services were used if required in order to educate patients in their preferred language. Less than 2% of our patients spoke other languages but English or Spanish.

Some studies report better outcomes in asthma with the use of the asthma action plan [3, 4]. Asthma action plan is highly recommended in addition to education in the GINA guidelines to improve outcomes in asthmatics. The Cochrane review found that self-management of asthma using a written action plan based on peak expiratory flow rates was equivalent to using symptoms based written action plan [19].

When patients with more than one visit to ER or more than one hospitalization incidence were evaluated, we found no outcome benefits from the education program. Possible explanations for the failure of education in this subgroup include (a) ineffective education sessions, (b) use of ER to obtain medications, (c) lack of “off hours” ambulatory services, (d) presence of comorbid conditions, and (e) lack of insurance. An additional asthma education session for reinforcement may be beneficial in these patients. There was no difference in clinical and baseline characteristics of this subgroup. The three most common comorbid conditions in our asthma cohort were psychiatric conditions, GERD, and OSA. In one study, 9.7% of asthmatics suffered from anxiety and 9% suffered from depression as compared to 6.6% and 5.5% prevalence, respectively, among nonasthmatic patients [20]. There is inconclusive data regarding the use of relaxation therapy to improve medication adherence, cognitive behavioral therapy to improve QoL, and use of biofeedback to improve peak expiratory flow in asthmatics [21].

Asthma Control Test is a well-validated tool to assess poorly controlled asthma as defined by GINA guidelines [22]. The ACT score is likely the simplest and widely used test to assess asthma control and evaluate the effectiveness of asthma education. Another method to evaluate asthma control is maintenance of a diary by the patient which can be tedious and patient compliance is a potential barrier. Mehuys et al. showed improved ACT scores after implementation of pharmacist-led asthma education program [23].

Improving asthma control and decreasing ER visits and hospitalization could have beneficial effect in healthcare cost and patient care. Yilmaz and Akkaya showed a decrease in ER visits and improved QoL scores in a small group of patients after six education sessions over one year [6]. Urek et al. compared the use of individual verbal instruction (three one-hour sessions) with written asthma information (asthma booklet) and with asthma group classes (four hours of group education) in 60 adults with moderate persistent asthma during a three-month follow-up. They reported that asthma group classes and individual verbal education improved QoL and asthma control [24].

There are many barriers in our inner city community for implementation of education sessions during prolonged period as used in other trials. Those potential barriers include financial and time constraints to return to clinic for education and perception of low value to education in disease management. In settings where education is provided solely by the physician, time constraints, lack of reimbursement, and proper training in education are potential barriers. Our study shows that a single education session linked to the physician visit has a positive impact on asthma control and healthcare utilization.

*Strengths*. This study evaluated the impact of an asthma education program as part of the physician visit in an inner city population with socioeconomic disadvantages. Implementation of a single session requires less institutional support and can be well and easily standardized and incorporated within physician visit. In our center, incorporation of a respiratory therapist and a clinical pharmacist helps us to fill other needs of patient care like performance of pulmonary functions test and medication evaluation and management. 

*Limitations*. These include limitations of a pre-post retrospective study including selection bias toward patients with difficult-to-control asthma or poor adherence to care. In addition, it is difficult to separate the effect of physician influence during the visit. Unless patients reported ER visits or hospitalization to other hospitals but BLHC, this data was not captured. In general, our population tends to seek care at their main hospital. Although correct use of inhaler was evaluated at the end of asthma education session, documentations by providers at clinical follow-up were inconsistent.

This study helps us to identify areas for improvement in the education program. These include routine asthma action plan and ACT score to be performed in all education sessions and need for further pulmonologist education regarding the value of education. This highlights the need for continued physician education and reinforcement in completion of all aspects of asthma education program by the educators.

### 4.1. Future Trend

The cost-effectiveness of asthma education programs has been evaluated by several researchers. They concluded that education was cost-effective if it was able to decrease hospital admissions and income lost because of asthma [25, 26]. Larger studies are needed to evaluate the cost-effectiveness of programs with different structures in various population settings. Focus in those patients with frequent ER visits or admissions will be welcome.

## 5. Conclusion

In the management of asthma, implementation of a single adult asthma education session as part of physician visit had a positive impact on asthma control and healthcare utilization. We suggest that models like this could be easily developed in a socially disadvantaged population. Carefully chosen educators could minimize institutional cost, as they could fill other gaps in the patient care.

## Figures and Tables

**Table 1:** Baseline clinical and demographic characteristics.

Characteristics	*n* = 231
Age, year (average ± SD)	53.28 ± 15.84
Gender, female	176 (76.2%)
*Race*	
Black	67 (29%)
Hispanic	115 (49.8%)
Tobacco, active cigarette smoker	57 (24.7%)
BMI ± SD	31.85 ± 7.13
*Severity of persistent asthma*	
Mild	55 (23.8%)
Moderate	124 (53.7%)
Severe	46 (19.9%)
*Comorbidities*	
Obstructive sleep apnea	34 (14.7%)
Pulmonary hypertension	23 (9.9%)
Gastroesophageal reflux	66 (28.6%)
Psychiatric illness on medication	72 (31.2%)
Congestive heart failure	23 (9.9%)
Ischemic heart disease	20 (8.6%)
Chronic renal disease	20 (8.6%)
Allergic rhinitis	9 (3.9%)
FEV1/FVC < 70	61/186 (32.8%)

**Table 2:** Main study outcomes.

Outcomes	Before education *n* = 231	After education *n* = 231	*p* value
Number of patients requiring ER visits	94	60	0.0005
Number of patients requiring hospitalization	49	26	0.0015
Average number of ER visits/patient/year	0.91 ± 1.748	0.61 ± 1.401	0.04
Average number of admissions/patient/year	0.34 ± 0.87	0.19 ± 0.70	0.0015
Average ICU admission/patient/year	0.03 ± 0.183	0.03 ± 0.195	0.7637
Number of patients with controlled asthma (ACT ≥ 20)	20 (*N* = 126)	44 (*N* = 126)	0.0001
Average number of clinic visits/patient/year	1.76 ± 2.51	2.18 ± 2.40	0.0185
Number of patients with >1 ER visits/year	41	31	0.7087
Number of patients with >1 admission/year	14	8	0.7202

## Abbreviations

ER: Emergency room
ACT: Asthma Control Test
PEFR: Peak expiratory flow rate
QoL: Quality of life.
